# Improvement of Electric Fish Optimization Algorithm for Standstill Label Combined with Levy Flight Strategy

**DOI:** 10.3390/biomimetics9110677

**Published:** 2024-11-06

**Authors:** Wangzhou Luo, Hailong Wu, Jiegang Peng

**Affiliations:** School of Automation Engineering, University of Electronic and Science Technology of China, Chengdu 611731, China

**Keywords:** Electric Fish Optimization algorithm, meta-heuristic algorithm, Levy flight, standstill label, local optimum

## Abstract

The Electric Fish Optimization (EFO) algorithm is inspired by the predation behavior and communication of weak electric fish. It is a novel meta-heuristic algorithm that attracts researchers because it has few tunable parameters, high robustness, and strong global search capabilities. Nevertheless, when operating in complex environments, the EFO algorithm encounters several challenges including premature convergence, susceptibility to local optima, and issues related to passive electric field localization stagnation. To address these challenges, this study introduces Adaptive Electric Fish Optimization Algorithm Based on Standstill Label and Level Flight (SLLF-EFO). This hybrid approach incorporates the Golden Sine Algorithm and good point set theory to augment the EFO algorithm’s capabilities, employs a variable-step-size Levy flight strategy to efficiently address passive electric field localization stagnation problems, and utilizes a standstill label strategy to mitigate the algorithm’s tendency to fall into local optima during the iterative process. By leveraging multiple solutions to optimize the EFO algorithm, this framework enhances its adaptability in complex environments. Experimental results from benchmark functions reveal that the proposed SLLF-EFO algorithm exhibits improved performance in complex settings, demonstrating enhanced search speed and optimization accuracy. This comprehensive optimization not only enhances the robustness and reliability of the EFO algorithm but also provides valuable insights for its future applications.

## 1. Introduction

In the mid-20th century, the establishment of bionics led scientists to seek new inspiration from living organisms. Consequently, many researchers developed bionic evolutionary algorithms, known as natural heuristic algorithms, based on the biological evolution mechanisms found in nature, to address complex real-life problems. Meta-heuristic algorithms, a broad category of heuristic algorithms, are particularly noteworthy as they do not depend on specific problem conditions and are applicable to a wide range of scenarios. Notable examples of meta-heuristic algorithms include Particle Swarm Optimization (PSO) and Genetic Algorithm (GA). Research on the optimization of meta-heuristic algorithms has consistently remained a prominent and dynamic area within the field. By optimizing meta-heuristic algorithms, significant improvements in algorithm performance can be achieved, particularly in tackling complex problems such as function combination optimization and function solving. This optimization is essential in meeting diverse application requirements [1].

With the deepening of biomimetics research, underwater biomimetics has become an emerging branch, focused on mimicking the characteristics of aquatic organisms, which is crucial for developing systems and structures that can operate efficiently in underwater environments. The navigation, foraging, and predator evasion behaviors of underwater organisms not only provide valuable design principles for underwater robots and autonomous underwater vehicles (AUVs), but also provide inspiration for the development of new underwater biomimetic algorithms. These algorithms provide rich strategies and tools for the design and optimization of underwater systems by simulating the adaptability and survival strategies of these organisms.

In this field, researchers have developed various algorithms inspired by the behavior of underwater animals. Kourepinis et al. proposed an artificial fish swarm optimization algorithm for the Urban Transit Routing Problem (UTRP) [2], achieving superior results in route set evaluation and direct trip coverage compared to 14 other algorithms on Mandl’s benchmark, with a new operator cost indicator providing insights on user-operator cost trade-offs. Zhao et al. introduced the Electric Eel Foraging Optimization (EEFO) algorithm [3], which models the foraging behaviors of electric eels to balance exploration and exploitation, outperforming many other algorithms in a variety of optimization problems. Braik et al. introduced the White Shark Optimizer (WSO) [4], a meta-heuristic algorithm inspired by the hunting and foraging behaviors of great white sharks, which effectively balances exploration and exploitation to achieve optimization in continuous search spaces and has been validated through rigorous benchmarking on CEC-2017 test functions and real-world problems. Hu et al. proposed the Genghis Khan Shark Optimizer (GKSO) [5], a novel meta-heuristic inspired by the shark’s predation and survival behaviors, demonstrating strong exploration and exploitation capabilities and outperforming other optimization algorithms on various benchmark functions and real-world engineering problems. Yildizdan enhanced the Artificial Jellyfish Search (AJS) algorithm by introducing modifications to both local and global search strategies in the Modified Jellyfish Search (MJS) algorithm [6], resulting in improved search capabilities and competitive performance on a wide range of continuous optimization problems. Jia et al. demonstrated the Crayfish Optimization Algorithm (COA) [7], which simulates crayfish behaviors to balance exploration and exploitation, showing good optimization effects in both benchmark functions and real-world engineering problems. Zhao et al. introduced the Manta Ray Foraging Optimization (MRFO) algorithm [8], which emulates the sophisticated foraging behaviors of manta rays, including chain, cyclone, and somersault strategies, to address complex engineering optimization problems, with a proven advantage in computational efficiency and solution accuracy. These algorithms improve the efficiency and effectiveness of the optimization process by simulating fish swarm behavior, predation strategies, and communication methods.

The Electric Fish Optimization (EFO) algorithm, proposed by Yilmaz and Sen in 2020, is another innovative achievement inspired by the predation behavior and communication methods of weak electric fish [9]. This meta-heuristic algorithm draws inspiration from the foraging behavior and communication patterns of weak electric fish. These fish, which inhabit muddy waters with poor visibility, face challenges in perceiving their surroundings due to limited visual capabilities, particularly in low-light conditions. Consequently, they have developed a unique skill known as electrical localization, which enables them to sense and navigate their environment effectively.

The Electric Fish Optimization (EFO) algorithm, known for its characteristics of few tunable parameters, strong robustness, and excellent global search ability, has garnered significant attention from researchers. Deepa and Madhavan developed the HREFSO algorithm by integrating the EFO algorithm with Rat Swarm Optimization (RSO) to optimize the selection threshold of skin lesion segmentation models [10]. Ibrahim et al. demonstrated the efficacy of the EFO algorithm in feature selection, particularly in handling complex optimization problems [11]. Kumar and Karri innovatively combined the EFO algorithm with Earthworm Optimization to reduce latency in cloud computing environments [12]. Notably, Rao and Madhu introduced a hybrid algorithm that merges the EFO algorithm with the Dragonfly Algorithm, enhancing the efficiency of large-scale MIMO systems [13]. Similarly, R. Anirudh Reddy and N. Venkatram devised a hybrid algorithm by integrating Horse Swarm Optimization (HOA) into the EFO algorithm for optimal path selection in network routing [14]. Moreover, Viswanadham and Jayavel proposed a novel approach using a hybrid EFO algorithm and Harris Eagle Optimization for data protection in supply chain networks [15]. Additionally, the EFO algorithm finds applications in diverse fields such as the classification of artificial neural networks [16], economic load scheduling [17], and connected and autonomous vehicle (CAV) technology [18].

In order to improve the efficiency of the Electric Fish Optimization (EFO) algorithm, this paper presents the Adaptive Electric Fish Optimization Algorithm Based on Standstill Label and Levy Flight (SLLF-EFO) to enhance the adaptability of the EFO to intricate environments. The proposed algorithm integrates strategies such as Levy flight, standstill label, and adaptive global range. These strategies effectively address the challenges faced by the algorithm and aim to improve its efficiency in various aspects, ultimately enabling it to find optima more effectively. The main contributions and innovations of this paper are as follows:

1. This paper optimizes the EFO algorithm in complex environments by utilizing optimization schemes. In this paper, we introduce an adaptive search range adjustment mechanism based on historical fitness values, which enables the algorithm to accelerate the convergence speed while maintaining the global search capability, which provides a new solution for the adaptability of the algorithm.
2. In the active electric field localization stage of the EFO algorithm, we optimize the individual selection logic by introducing the distance threshold, which avoids the rapid aggregation of the algorithm in the early stages, so as to enhance the exploration ability of the algorithm.
3. In this paper, the golden sine operator is innovatively integrated in the passive electric field localization stage, and this strategy uses the golden ratio coefficient to guide the search steps and directions, which further improves the local search ability of the EFO algorithm.
4. Much of the existing research on meta-heuristic algorithm optimization focuses on avoiding local optima, without considering how to enable the algorithm to escape local optima once trapped. In addition to the systematic optimization strategies mentioned above, the SLLF-EFO algorithm proposed in this paper innovatively adopts a standstill label strategy to optimize the iterative process after becoming stuck in a local optimum. This strategy allows the algorithm individuals to actively escape after identifying themselves as trapped in a local optimum, thereby indirectly improving the algorithm’s overall global search ability.
5. During multiple experimental studies on the EFO algorithm, it was found that due to the randomness of the EFO algorithm’s values, there is a small probability that all the electric fish in the algorithm will be in passive electric field localization mode. Passive electric field localization requires the participation of individuals who use active electric field localization, but there are no individuals in the population with active electric field localization at this time, which causes the algorithm to suddenly interrupt or stop running, a phenomenon referred to as passive electric field localization stagnation in this paper. Although the probability of this situation occurring is extremely small, this paper introduces a variable-step-size Levy flight strategy to address it, which not only improves the global search ability of the algorithm but also resolves the passive electric field localization stagnation problem mentioned above.

## 2. Electric Fish Optimization Algorithm and Its Problems

Across various practical applications, meta-heuristic algorithms like Electric Fish Optimization (EFO) are widely utilized to tackle optimization challenges. These challenges are encapsulated by an objective function $y=F(x_{1},x_{2},\cdots ,x_{N})$, which assesses the performance or fitness of solutions across different combinations of decision variables $x_{1},x_{2},\cdots ,x_{N}$. The function’s output, *y*, serves as the fitness value, indicating the solution’s effectiveness.

To maintain solution feasibility, interval constraints are essential: $x_{minj}\leq x_{j}\leq x_{maxj}$ for j=1,2,⋯,N. These bounds define the permissible range for each decision variable, ensuring the search remains within practical limits and that the solutions found are actionable.

Within this meta-heuristic framework, algorithmic individuals represent potential values for the decision variables. The algorithm aims to identify, through iterative processes, a set of values for $x_{1},x_{2},\cdots ,x_{N}$ that maximizes or minimizes the objective function *y*. This iterative evaluation of the objective function for different variable sets allows the algorithm to converge towards the optimal solution. The EFO algorithm leverages the foraging and communication behaviors of weak electric fish to develop an innovative search strategy. This approach equips it to efficiently address complex optimization problems, seeking optimal or near-optimal solutions within predefined interval constraints.

### 2.1. Electric Fish Optimization Algorithm

The Electric Fish Optimization algorithm divides the population into active and passive electro-located electric fish based on the magnitude of the electric field frequency of the weak electric fish. The active electro-located electric fish can be used to search for food and avoid danger within a limited range through the electric signals they generate, while the passive electro-located electric fish can be used for electro-location through receiving electric signals [9]. The design of the EFO algorithm mainly consists of three parts: population initialization, active electrical positioning, and passive electrical positioning.

#### 2.1.1. Population Initialization

In the EFO algorithm, the core parameters of electric fish individuals are their own position, anad the frequency and amplitude of their electrical signals. The population initialization adopts a random initialization scheme, which randomly generates the coordinate values of *N* electric fish individuals in the *D*-dimensional space.

After initializing the position, each electric fish sets the frequency value of its electrical signal according to its own position. The frequency value $f_{i}^{t}$ of the *i*-th individual in the algorithm at the *t*-th iteration is determined by the individual’s fitness value, that is,

(1) $$f_{i}^{t}=f_{min}+\left(\frac{{fit}_{worst}^{t}-{fit}_{i}^{t}}{{fit}_{worst}^{t}-{fit}_{best}^{t}}\right)\left(f_{max}{-f}_{min}\right)$$

In Equation (1), ${fit}_{i}^{t}$ is the fitness value of the *i*-th individual at the *t*-th iteration; ${fit}_{worst}^{t}$ and ${fit}_{best}^{t}$ represent the worst and best fitness values of the electric fish population at the *t*-th iteration, respectively; $f_{min}$ and $f_{max}$ are the minimum and maximum values of frequency, respectively. In the algorithm, this frequency is mainly used for probability calculation, so $f_{max}=1$ and $f_{min}=0$ are set.

The amplitude value of an individual determines the movable range of active electric field localization for electric fish, and also determines the probability of being perceived by passive-electric-field-localization electric fish. In the EFO algorithm, the amplitude value $A_{i}^{t}$ of the *i*-th individual at the *t*-th iteration is determined by the amplitude value $A_{i}^{t-1}$ from the previous iteration and the frequency value $f_{i}^{t}$ at this time. The calculation formula is as follows:

(2) $$A_{i}^{t}=\alpha A_{i}^{t-1}+\left(1-\alpha \right)f_{i}^{t}$$

In Equation (2), α is a constant within the interval [0,1], which determines the weight of the previous amplitude and the current frequency. The initial amplitude value of the *i*-th individual is equal to its current frequency value $f_{i}^{t}$. In this study, we set α to 0.9.

In the EFO algorithm, after each iteration, individuals with higher frequency values $\left(N_{A}\right)$ use active electric field localization (active mode), while individuals with lower frequency values $\left(N_{P}\right)$ perform passive electric field localization (passive mode) $\left(N_{A}+N_{P}=N\right)$. Afterwards, each individual moves in parallel according to their own situation to find the global optimum.

#### 2.1.2. Active Electric Field Localization

In the EFO algorithm, the range of active electric field localization is limited, so active electric field localization determines the local search ability of the EFO algorithm. The range of motion of the *i*-th individual in active mode is determined by its current amplitude value $A_{i}$, and its calculation formula is shown in Equation (3):

(3) $$r_{i}=\left(x_{maxj}-x_{minj}\right)A_{i}$$

The distance between the *i*-th and *k*-th individuals is determined by the Cartesian distance formula:

(4) $$d_{ik}=\left\|x_{i}-x_{k}\right\|=\sqrt{\sum_{j=1}^{D}{\left(x_{ij}-x_{kj}\right)}^{2}}$$

Afterwards, if there are no other individuals within the search range of the active individual, then the active individual performs Brownian motion within the active electric field range, as shown in Equation (5). If there are other individuals present, the individual randomly selects one active individual for position update, as shown in Equation (6).

(5) $$x_{ij}^{cand}=x_{ij}+\varphi \left(x_{kj}-x_{ij}\right)$$

(6) $$x_{ij}^{cand}=x_{ij}+\varphi r_{i}$$

In Equations (5) and (6), *k* is a randomly selected individual from other individuals within the search range, φ is a uniformly distributed random number within the interval [−1,1], and $x_{ij}^{cand}$ represents the candidate position for individual *i*.

#### 2.1.3. Passive Electric Field Localization

Unlike active electric field localization, passive electric field localization does not depend on the number of other active-mode individuals around individual *i*, and its movable range is much larger than the range of active electric field localization. Therefore, in the EFO algorithm, passive electric field localization determines the algorithm’s global search ability.

The probability that individual $k(k\in N_{A})$ in active mode is perceived by individual $i(i\in N_{P})$ in passive mode, $p_{k}$, is related to the individual amplitude values of all active modes and the distance between individual *i* and individual *k*. It is calculated using roulette wheel selection, the formula of which is as follows:

(7) $$p_{k}=\frac{A_{k}/d_{ik}}{\sum_{j\in N_{A}}A_{j}/d_{ij}}$$

According to the probability value obtained from Equation (7), K individuals are selected from $N_{A}$, their reference position $x_{rj}$ is determined using Equation (8), and a new position $x_{ij}^{new}$ is generated using Equation (9).

(8) $$x_{rj}=\frac{\sum_{k=1}^{K}A_{k}x_{kj}}{\sum_{k=1}^{K}A_{k}}$$

(9) $$x_{ij}^{new}=x_{ij}+\varphi \left(x_{rj}-x_{ij}\right)$$

In this logic, it is still possible for individuals with higher frequencies to perform passive electrical localization. When this situation occurs, the individual completely loses their location information. To avoid this situation, the EFO algorithm considers using Equation (10) to update the candidate position of individual *i* in the *j*-th dimensional search space. Among them, ${rand}_{j}\left(0,1\right)$ is a random number uniformly distributed in the (0,1) interval.

(10) $$x_{ij}^{cand}=\left\{\begin{array}{cc} x_{ij}^{new}, & {rand}_{j}\left(0,1\right)>f_{i}\\ x_{ij}, & else \end{array}\right.$$

The final step in passive electric field localization is to use Equation (11) to modify the candidate position of individual *i* in the *j*-th dimensional search space, in order to increase the diversity of the population.

(11) $$\begin{array}{c} x_{ij}^{cand}=x_{minj}+\varphi \left(x_{maxj}-x_{minj}\right)\\ {rand}_{1}\left(0,1\right)\leq {rand}_{2}\left(0,1\right) \end{array}$$

In the process of individual population localization, if the generated candidate position coordinates in a certain dimension exceed the specified global search range, the boundary coordinates of that dimension are considered as the candidate position coordinates to avoid going out of bounds, as shown in Equation (12).

(12) $$x_{ij}^{cand}=\left\{\begin{array}{cc} x_{maxj}, & x_{ij}^{cand}>x_{maxj}\\ x_{minj}, & x_{ij}^{cand}<x_{minj}\\ x_{ij}^{cand}, & otherwise \end{array}\right.$$

After processing, the EFO algorithm performs movement, and individuals compare the fitness values of the candidate position with the current position. If the fitness of the candidate position is better than the current position, the individual moves to the candidate position, otherwise they do not move.

**Figure 1:**
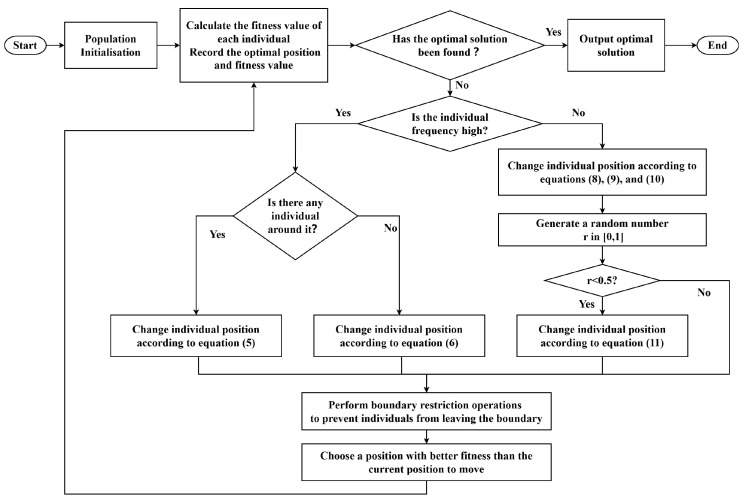
Basic process of Electric Fish Optimization algorithm.

The basic process of the EFO algorithm is shown in Figure 1. This figure illustrates the workflow of the EFO algorithm, including population initialization, active electric field localization, and passive electric field localization. It highlights how individuals in the population switch between active and passive modes based on their frequency values, guiding the optimization process. Based on the active/passive electric field localization logic of the EFO algorithm, it can be concluded that the active electric field localization of the EFO algorithm enables active-mode individuals to approach other active-mode individuals, while passive-mode individuals rely on passive electric field localization to approach the areas where active-mode individuals gather, reflecting the aggregation behavior of EFO algorithm individuals.

### 2.2. Problems Encountered by EFO

When facing high-dimensional complex environments where the number of dimensions of an individual or the number of extreme values in the search environment is gradually increased, the optimization performance of the EFO algorithm is significantly reduced. The main problems that arise due to these limitations are as follows.

1. Premature convergence: The characteristic of many heuristic algorithms, including the EFO algorithm, is to choose a better position to move to than the current one. In complex environments, the individual’s reference position may not be better than the current position. At this time, the individual will not move, resulting in multiple ineffective iterations and a significant decrease in algorithm efficiency, which is known as premature convergence [19,20,21].
2. Easy plunges into local optimum: Trapping in local optima is a common problem faced by most heuristic algorithms, and once trapped in a local optimum, individuals find it difficult to escape [22,23]. Due to the clustering trend of electric fish, the EFO algorithm is also susceptible to this problem, reducing its optimization ability.
3. Passive electric field localization stagnation: According to Equations (7) and (8), it can be inferred that the passive electric field localization operation of the EFO algorithm needs to refer to the position of the active individual in the field. During the operation of the EFO algorithm, there may be a situation where the electrical signal frequency of all individuals is very low, and all individuals are in passive mode. In this case, if there are no active-mode individuals on the field, it is difficult for all passive individuals to move and the algorithm comes to a standstill. After testing, it was found that the probability of this situation occurring is very small; it is referred to as the passive electric field localization stagnation problem in this article.

## 3. The SLLF-EFO Algorithm Proposed in This Article

**Figure 2:**
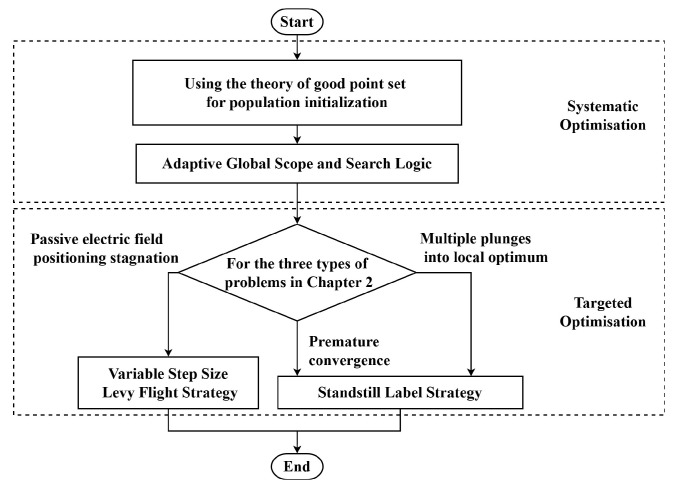
Optimization plan for Electric Fish Optimization algorithm.

The Adaptive Electric Fish Optimization Algorithm Based on Standstill Label and Levy Flight (SLLF-EFO) proposed in this paper is an optimization scheme for the EFO algorithm which incorporates standstill label and Levy flight strategies. This scheme includes both systematic and targeted optimizations for addressing the three aforementioned problems, thereby enabling the EFO algorithm to maintain high efficiency in diverse environments. The overall optimization strategy is illustrated in Figure 2. This figure presents the various strategies incorporated into the SLLF-EFO algorithm to enhance its performance. In terms of systematic optimization, good point set theory for population initialization, adaptive global scope, individual selection optimization, and the golden sine operator were used to improve the speed of the EFO algorithm. In terms of targeted optimization, the variable-step-size Levy flight strategy and the standstill label strategy methods were used to solve the three problems mentioned in Section 2.2, which not only enabled the EFO algorithm to jump out of local optima, but also improved its global search ability.

### 3.1. Population Initialization

This article introduces a theory called good point set theory in the EFO algorithm for population initialization, rather than choosing traditional random schemes. The concept of good point sets was initially proposed by Italian economist Pareto, and further developed by mathematician Hua Luogeng on this basis [24]. Compared to random point sets, good point sets provide better coverage of the entire search space and are used to optimize various meta-heuristic algorithms [25,26].

Let $G_{D}$ be a unit cube in a *D*-dimensional Euclidean geometric space, if the position $r\in G_{D}$, then for $P_{n}\left(i\right)=(r_{1}^{\left(n\right)}\cdot i,r_{2}^{\left(n\right)}\cdot i,\cdots ,r_{D}^{\left(n\right)}\cdot i),1\leq i\leq n$, its deviation $\varphi \left(n\right)=C\left(r,\varepsilon \right)n^{-1+\varepsilon }$, where C(r,ε) is a constant related only to *r*,ε, then the set of good points is called $P_{n}\left(i\right)$ and *r* is the good point.

In this paper, we take $r_{i}=2\cos(\frac{2\pi i}{p}),1\leq i\leq D$, where *p* is the smallest prime number that satisfies $\frac{p-3}{2}\geq D$.

**Figure 3:**
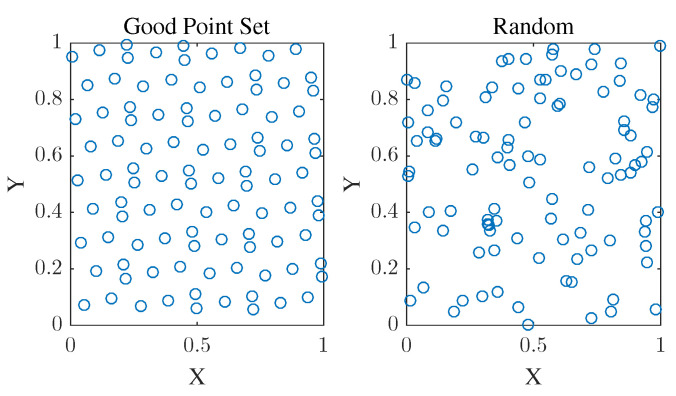
Population distribution of the good point set method and random method.

In order to evaluate the effectiveness of the best point set method, 100 initial population positions were created in the 2D plane using both the best point set and random placement methods. The results are shown in Figure 3. The left panel shows the initial population distribution using the good point set method, which is more uniform. The right panel shows the distribution using the random method. The results indicate that the initialization population distribution generated using the best point set method is more uniform, which can enhance the global search ability of the EFO algorithm to a certain extent.

### 3.2. Adaptive Global Scope and Search Logic

#### 3.2.1. Adaptive Global Scope

To improve the convergence speed of the algorithm, one can consider reducing the global range during the iteration process. However, in the early iterations of the algorithm, more new areas need to be discovered by the population, and it is not advisable to narrow the global scope at this time, as it will hinder the search for the global optimal value. Therefore, this article proposes the Adaptive Global Scope method, which is a dynamic adjustment method for the search range of the algorithm. Based on the historical worst fitness value, it determines when to shrink the global range and improve the convergence speed of the algorithm. This article determines the time point to initiate contraction based on the historical worst fitness values obtained in the experiment, which improves the efficiency of the region search process. Based on experimental results, it has been observed that setting the opening condition to one-tenth of the historical worst fitness value yields better performance in typical scenarios. The specific process of updating the global upper bound $Ulim_{j}$ and lower bound $Llim_{j}$ for the *j*-th dimension under this strategy is as follows:

(13) $$Llim_{j}=\left\{\begin{array}{cc} Llim_{j}, & all_{bst}>\frac{all_{wst}+9fit_{bst}}{10}\\ minX_{j}, & else \end{array}\right.$$

(14) $$Ulim_{j}=\left\{\begin{array}{cc} Ulim_{j}, & all_{bst}>\frac{all_{wst}+9fit_{bst}}{10}\\ maxX_{j}, & else \end{array}\right.$$

In Equations (13) and (14), $X_{j}$ refers to the *j*-th dimensional set of coordinates of all individuals, $all_{bst}$ and $all_{wst}$ refer to the global historical best fitness value and historical worst fitness value of this experiment, respectively. And, $fit_{bst}$ refers to the global optimal solution fitness value in this environment.

#### 3.2.2. Optimization of Individual Selection Strategy for Active Electric Field Localization

In the active electric field localization stage of the EFO algorithm, the active electric field localization individual detects other active individuals and randomly selects one to move. This article considers modifying this selection logic to select the closest individual for movement. This logic will lead to rapid population aggregation in the early iterations, as well as difficulty in escaping from local optima, which is not conducive to the algorithm’s search. Therefore, far and near thresholds are set up, so that the active individuals do not use the nearest-distance moving scheme in the above cases, and switch to the random selection scheme of the original EFO algorithm. Experimental tests show that using the value of the individual’s dimension *D* to set the distance threshold is more effective; the far distance threshold $d_{MaxLim}$ and near threshold $d_{MinLim}$ satisfy Equation (15):

(15) $$\left\{\begin{array}{c} d_{MaxLim}=10^{-12}\times \sqrt{D}\\ d_{MinLim}=10^{-17}\times \sqrt{D} \end{array}\right.$$

#### 3.2.3. Introduction of Golden Sine Operator

The Golden Sine Algorithm (GSA) is an optimization algorithm based on the sine function and the golden section coefficient [27]. The algorithm was originally proposed by Erkan Tanyildizi et al. in 2017 [27]. The design of the algorithm is inspired by the sine function in mathematics, and the sine function for computational iterative optimization search has the advantages of fast convergence speed, good robustness, ease of implementation, and having fewer parameters and operators to regulate; thus, it is used in different fields of problem solving [28,29].

GSA introduces the golden ratio coefficient in the position update process, allowing GSA to fully search the region, which produces excellent solutions in each iteration process, rather than the entire region, improving the algorithm’s optimization speed and possessing strong local search ability. The formula for updating individual positions in the Golden Sine Algorithm is

(16) $$V_{ij}^{t+1}=V_{ij}^{t}|\sin\left(r_{1}\right)|+r_{2}\cdot \sin\left(r_{1}\right)\left|u_{1}P_{ij}^{t}-u_{2}V_{ij}^{t}\right|$$

In Equation (16), $V_{ij}^{t}$ denotes the current position of individual *i* in the *j*-th dimension; $P_{ij}^{t}$ denotes the optimal position of all individuals in the *j*-th dimension of the optimal position; $V_{ij}^{t+1}$ represents the new position of individual *i* in the *j*-th dimension; $r_{1}\in \left[0,2\pi \right]$ and $r_{2}\in \left[0,\pi \right]$ determine the search step and search direction, respectively, and both obey a normal distribution; $u_{1}$ and $u_{2}$ are adjustable parameters used to control the search space, and they satisfy the following relationship:

(17) $$u_{1}=a\cdot \tau +b\cdot \left(1-\tau \right)$$

(18) $$u_{2}=a\cdot \left(1-\tau \right)+b\cdot \tau$$

In Equations (17) and (18), τ is the golden section coefficient $\frac{\sqrt{5}-1}{2}$. In order to continuously narrow down the search scope, the values of *a* and *b* are also adjusted according to the location of the individual, and the initial values of *a* and *b* are, respectively, −π and π.

In this paper, the golden sine operator is introduced in the last step of the passive electric field localization stage to randomly select the position of one dimension of an individual for updating. The optimized position update formula is shown below.

(19) $$x_{ij}^{cand}=x_{ij}|\sin\left(r_{1}\right)|+r_{2}\cdot \sin\left(r_{1}\right)\left|u_{1}x_{pj}-u_{2}x_{ij}\right|\;{rand}_{1}\left(0,1\right)\leq {rand}_{2}\left(0,1\right)$$

In Equation (19), $x_{pj}$ refers to the current position of the best individual in the *j*-th dimension of the population. To simplify the iterative process and improve the computational efficiency, in this paper we set the coefficients $u_{1}$ and $u_{2}$ to fixed values as follows:

(20) $$u_{1}=-\pi \cdot \tau +\pi \cdot \left(1-\tau \right)$$

(21) $$u_{2}=-\pi \cdot \left(1-\tau \right)+\pi \cdot \tau$$

### 3.3. Variable-Step-Size Levy Flight Strategy

#### 3.3.1. Levy Flight Strategy

In this article, we consider introducing the Levy flight strategy to address problem (3) discussed in Section 2.2. Levy flight, named after French mathematician Paul Levy and first proposed by Benoit Mandelbrot in 1982, is a special type of stochastic flight characterized by a heavy-tailed probability distribution of step size [30]. It deviates from traditional random walk processes such as Brownian motion by giving individuals a higher likelihood of moving over long distances, leading to increased uncertainty. Due to its heavy-tailed distribution, Levy flight can better simulate natural random movement and is widely used in various meta-heuristic algorithms to optimize performance [31,32,33].

**Figure 4:**
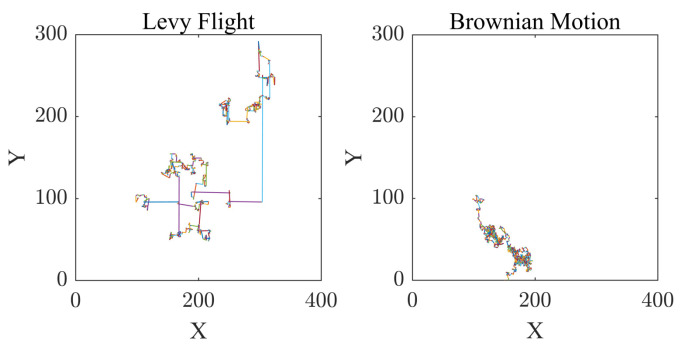
Random walk trajectories of Levy flight and Brownian motion.

Figure 4 shows the results of 1000 Levy flights and 1000 Brownian motions performed by a single individual on a 2D plane. The left panel illustrates the trajectory of 1000 Levy flights, characterized by longer, sporadic jumps, enhancing the exploration capability. The right panel shows the trajectory of 1000 Brownian motions, characterized by shorter steps, which reflects the characteristic that Brownian motion can only search within a local range. From the figure, it can be seen that Levy flight relies on its high probability of long-distance flight to obtain a larger search range, which can improve the ability of individuals in the algorithm to discover new regions in applications.

Due to the extremely high complexity of Levy flight, the Mantegna algorithm is commonly used to achieve a symmetric Levy-stable distribution [34]. The probability density function of Levy flight step size under the Mantegna algorithm can be expressed as Equation (22):

(22) $$\left\{\begin{array}{c} \begin{array}{cc} L\left(s\right)\;{\left|s\right|}^{-1-\beta }, & 0<\beta \leq 2 \end{array}\\ \begin{array}{cc} s=\frac{\mu }{{\left|v\right|}^{\frac{1}{\beta }}} & \; \end{array} \end{array}\right.$$

In Equation (22), *s* is the random step size, β is the distribution parameter, and μ and *v* are all random numbers within the interval [0,1], obeying a normal distribution within the interval.

By integrating Levy flight into the EFO algorithm, an individual will engage in Levy flight movement when passive electric field localization fails to detect any active-mode individual. The introduction of Levy flight significantly broadens the activity range of the electric fish. This breakthrough not only breaks the stagnation phenomenon of the algorithm but also enhances the individual’s capacity to explore previously uncharted territories.

#### 3.3.2. Variable-Step-Movement Logic

Experimental tests have shown that the aggregation behavior of the EFO algorithm causes most individuals to converge to the global optimum in the later iterations. However, if the Levy flight strategy is used again at this time, individuals may deviate from the global optimum due to long-distance movement. To alleviate this problem, this article considers adjusting the step size range of Levy flight at different times during the whole iteration process.

The logistic model was proposed in 1837 by Verhulst, a German biologist, in his study of the laws of biological reproduction [35,36]. It is widely used to describe the process of saturation, known as the “S” process, and the logistic model’s function curve, also referred to as the “S” curve, represents a gradual growth deceleration and stabilization phenomenon. The general function expression of the logistic model is as follows:

(23) $$f=\frac{\alpha }{1+e^{\beta -\gamma x}}$$

To introduce the logistic model into the step size limitation strategy of Levy flight, it is necessary to integrate the fitness function of the EFO algorithm and determine the distance range of this Levy flight based on the current individual’s fitness value. The solution is to normalize the fitness values of individual *i* based on the best and worst historical fitness values of the population, in order to obtain the normalized fitness ${fit}_{i}^{nom}$, in order to import the logistic model.

For this, the logistic model function variables are adjusted and the normalized fitness values are substituted into them; when substituting, it is necessary to perform a scaling transformation on ${fit}_{i}^{nom}$ to map it to the region range of the logistic model [−10, 10]. However, when individuals approach local optima, their ${fit}_{i}^{nom}$ are often small, which leads to a smaller step size for Levy flight and hinders individuals from escaping local optima. To address this issue, this article introduces a threshold. If the current normalized fitness of an individual is below this threshold, the step size range of Levy flight will be determined based on the number of iterations at that time. The experiment shows that the effect is better when the threshold is set to $10^{-4}$, and the final step length formula is shown in Equation (24), where itercount is the current iteration number.

(24) $${step}_{i}=\left\{\begin{array}{cc} \frac{1/25}{1+e^{10-20{fit}_{i}^{nom}}}, & {fit}_{i}^{nom}>10^{-4}\\ \frac{1}{\sqrt{itercount}}, & else \end{array}\right.$$

In the end, the *i*-th individual performs a Levy flight with the logic of

(25) $$x_{ij}^{new}=x_{ij}+{step}_{i}\cdot sign\left(rand-0.5\right)⊗Levy$$

In Equation (25), $sign\left(rand-0.5\right)$ is the sign function, which is used to adjust the direction of the Levy flight, and it can enhance the randomness of the search and avoid falling into a local optimum. ⊗ is the point-to-point multiplication, Levy follows Equation (22).

### 3.4. Standstill Label Strategy

Issues 1 and 2 in Section 2.2 highlight a prevalent challenge known as stagnation, wherein individuals remain stationary for prolonged periods. Stagnation represents a common obstacle encountered by heuristic algorithms. While existing EFO optimization methods aim at preventing stagnation, strategies for addressing this state post-occurrence have not been thoroughly explored. To tackle the aforementioned challenges, a standstill label strategy is introduced in this paper as a novel approach.

The standstill label strategy originates from the living habits of ant colonies. When ant colonies explore, they leave pheromones on the paths they have already walked, guiding or reminding other individuals to follow up or change direction for search; moreover, when individuals move to hazardous areas, warning pheromones are also released. When ant colony individuals recognize this pheromone, they will not approach or immediately move away from that location, which can greatly improve the search efficiency of the population [37].

The SLLF-EFO algorithm proposed in this article considers using an iteration counting method to determine whether the algorithm has fallen into a stagnant state of a local optimum. After each iteration of the population search, the algorithm detects whether individuals in the population have moved. If the individual does not move after the iteration, the counter increments by 1, otherwise it resets to 0. When the counter reaches a certain value, the individual is considered to be in a stagnant state. In this study, we set the counting threshold of this counter to 50.

Obviously, if each individual in the population is assigned its own independent counter, it will significantly increase the computation time of the algorithm. Fortunately, due to the aggregation behavior of EFO, most individuals tend to gather together during the counting process. Due to the close proximity of individuals, it is feasible to determine the current behavior state of the group by the position of the individual with the best fitness value at this time in order to improve the computational efficiency of the algorithm. Therefore, simply setting up a counter to observe the stagnant state of the current global optimal individual can be regarded as the running state of the entire population, which can greatly simplify the complex calculation process of counting each individual separately and improve the running speed of the algorithm.

The standstill label strategy proposed in this article stipulates that when the best fitness individual is detected to be in a stagnant state, all individuals in the population are forced to perform a variable-step-size Levy flight operation once, and this variable-step-size Levy flight operation is not constrained by the global scope and environmental fitness; that is, individuals are allowed to move outside the current global scope or to a position with a fitness worse than their current location. After Levy flight, the algorithm updates the global scope again according to the current population distribution. This not only frees all individuals from local optima, but also avoids the problem of the global optimum not being within the global scope due to global shrinkage. And before moving, the algorithm leaves a pheromone at the global optimal individual position (stagnation point) as a label. When an individual moves to the stagnation point again, there is no need to perform the aforementioned counting operation. Instead, they can directly perform the variable-step-size Levy flight operation to escape and update the global range again, so that the individual immediately leaves the stagnation point. This can effectively reduce the number of subsequent invalid iterations and improve the population’s ability to find the global optimum.

### 3.5. Complexity of SLLF-EFO Algorithm

In order to comprehensively evaluate the performance of the improved SLLF-EFO algorithm, in this section a detailed analysis of the time complexity of the original EFO algorithm and the SLLF-EFO algorithm is conducted. Time complexity is an important indicator for measuring algorithm efficiency, reflecting the trend in algorithm execution time as the input size increases.

The time complexity analysis of the EFO algorithm is based on the following steps:

1. Population initialization: Randomly assign positions to each individual in the search space, with a time complexity of O(N×D), where *N* is the population size and *D* is the dimension of the search space.
2. Main loop process: During this process, individuals in the EFO algorithm use either active or passive electric field localization for movement operations, both of which may involve interactions between each individual and several other individuals in the population. Considering the worst-case scenario, where each individual in an iteration interacts with all other individuals in the population once, the time complexity of this stage is $O(N^{2}\times D)$.
3. Termination condition check: Check whether the algorithm meets the termination condition after each iteration. This is a constant time operation with a time complexity of O(1).

If the upper limit of iteration times is $Iter_{max}$, then the time complexity of the EFO algorithm is as shown in Equation (26) [11].

(26) $$\begin{array}{cc} T(\mathrm{EFO}) & =O\left(N\times D\right)+Iter_{max}\times O\left(N^{2}\times D\right)+O\left(1\right)\\ \; & =O(Iter_{max}\times N^{2}\times D) \end{array}$$

The enhancement mechanism introduced by the SLLF-EFO algorithm did not significantly change the time complexity, therefore its time complexity is similar to that of the EFO algorithm. Specifically, as follows.

1. Population initialization: Using good point set theory for population initialization, the time complexity of this step is still O(N×D).
2. Main loop process: For active electric field localization, this article introduces a strategy of adaptive global scope and search logic, which dynamically adjusts the global range without increasing complexity; the introduction of the distance threshold modifies the active electric localization search logic of individuals, but does not change the number of times individuals need to communicate with other individuals in the population in the original EFO algorithm. Therefore, the complexity of the active electric localization stage is still $O(N^{2}\times D)$. And, for passive electric field localization, the golden sine operator we adopt only modifies the reference position of the individual, without modifying the wheel selection of the passive individual; moreover, the use of variable-step-size Levy flight strategy is also a solution to the passive electric field localization stagnation problem that occurs with extremely low probability, and its proportion in the iterative process can be ignored. Therefore, the complexity of passive electric localization in the SLLF-EFO algorithm is also the same as that in the EFO algorithm.
3. Standstill label strategy: This strategy is activated when the optimal individual is identified as being in a stagnant state. It involves performing a variable-step-size Levy flight operation, and the time complexity of this part is O(N×D).
4. Termination condition check: Similar to the EFO algorithm, the SLLF-EFO algorithm checks whether the algorithm meets the termination condition after each iteration. The time complexity here is still O(1).

So the time complexity calculation of the SLLF-EFO algorithm is shown in Equation (27). In the formula, $K_{s}$ refers to the number of times the stagnation flag strategy is enabled in $Iter_{max}$ iterations, and in general, $K_{s}$ will be much smaller than $Iter_{max}$.

(27) $$\begin{array}{cc} T(\text{SLLF-EFO}) & =O\left(N\times D\right)+Iter_{max}\times O\left(N^{2}\times D\right)+K_{s}\times O\left(N\times D\right)+O\left(1\right)\\ \; & =O(Iter_{max}\times N^{2}\times D) \end{array}$$

By comparing the time complexity analysis of the SLLF-EFO algorithm and the original EFO algorithm, we found that both have the same time complexity, that is, $O(Iter_{max}\times N^{2}\times D)$. This result indicates that although the SLLF-EFO algorithm introduces various improvement mechanisms to enhance its search ability and robustness in complex environments, these improvements have not led to an increase in the overall computational cost of the algorithm. This indicates that the SLLF-EFO algorithm improves optimization performance and solution quality by implementing more effective search strategies and avoiding local optima while maintaining the same computational efficiency as the original algorithm. Therefore, the SLLF-EFO algorithm not only has higher optimization efficiency in theory, but is also more likely to find better solutions in practical applications.

## 4. Experiments Based on Benchmark Functions

### 4.1. Test Functions and Comparison Algorithms

Due to the limitations of NP theory, it is difficult to prove the strict convergence of meta-heuristic algorithms. In most cases, meta-heuristic algorithms can only find approximate optimal values [38,39]. Therefore, many discussions on the convergence and optimization quality of meta-heuristic algorithms are verified through experimental testing using standard test functions or a certain application scenario. The optimization performance of the algorithm is comprehensively obtained through comparative experiments in multi-algorithm and multi-function environments [40,41,42]. This article also adopts the method of using standard test functions to intuitively evaluate the optimization performance of the SLLF-EFO algorithm.

In order to visually evaluate the optimization performance of the SLLF-EFO algorithm, this paper compares it with four algorithms in Python, including the original EFO algorithm and three classical meta-heuristic algorithms: the Particle Swarm Optimization (PSO) algorithm [43], Differential Evolution (DE) [44], and Genetic Algorithm (GA) [45]. The evaluation was performed using Python 3.11, with an AMD Radeon(TM) R5-5500U Graphics processor, with the Windows 11 operating system as the computational platform. Comparing with classical algorithms can fully demonstrate the optimization effect of the SLLF-EFO algorithm.

Twelve CEC benchmark test functions, depicted in Table 1, were chosen to assess the adaptability of the SLLF-EFO algorithm to intricate environments. The functions selected for this study can be referenced in [46,47]. Among them, F1 to F4 are high-dimensional single-peak functions, F5 to F7 are high-dimensional multi-peak functions, F8 and F9 are low-dimensional single-peak functions, and F10 to F12 are low-dimensional multi-peak functions.

After parameter tuning through experimental testing, we set the relevant parameters of each selected algorithm as follows: The learning factors in the PSO algorithm used for experiments $c_{1}$, $c_{2}$ are set to 2; the maximum value of the inertia weight ω is 0.9 and the minimum value is 0.4; in the DE algorithm, the scaling factor is set to 0.9 and the crossover factor CR is 0.6; the GA algorithm used has a crossover probability of 0.6 and a mutation probability of 0.05. Under the parameter conditions set in this article, each algorithm can achieve excellent performance under the selected function.

To ensure the fairness of the experiments, all algorithms use the same parameters, their population size is set to 30 individuals, and the upper limit on the number of iterations for each algorithm is set to 2000. In addition, each individual in all algorithms has only one evaluation of the objective function in each iteration. Therefore, when the number of individuals in the population is the same, we can use the number of iterations as an effective indicator to evaluate the speed of the algorithm. Moreover, since four algorithms are based on random search optimization algorithms, there is a certain degree of chance in the results of a single experiment. In order to avoid this situation and further reflect the robustness of the algorithms, each algorithm is run independently 100 times on each benchmark function, and the minimum number of iterations (Min-Iter), the maximum number of iterations (Max-Iter), the average number of iterations (Mean-Iter), and the number of times the iteration limit is exceeded (Over-Num) are taken as the four indicators of the convergence speed of the algorithms. To reflect the convergence accuracy of the algorithm, the best value (Min), the worst value (Max), the average value (Mean) and the standard deviation (Std) of the 100 experimental results are taken as the indexes for evaluating the search accuracy of them.

### 4.2. Speed Comparison with Other Algorithms

**Figure 5:**
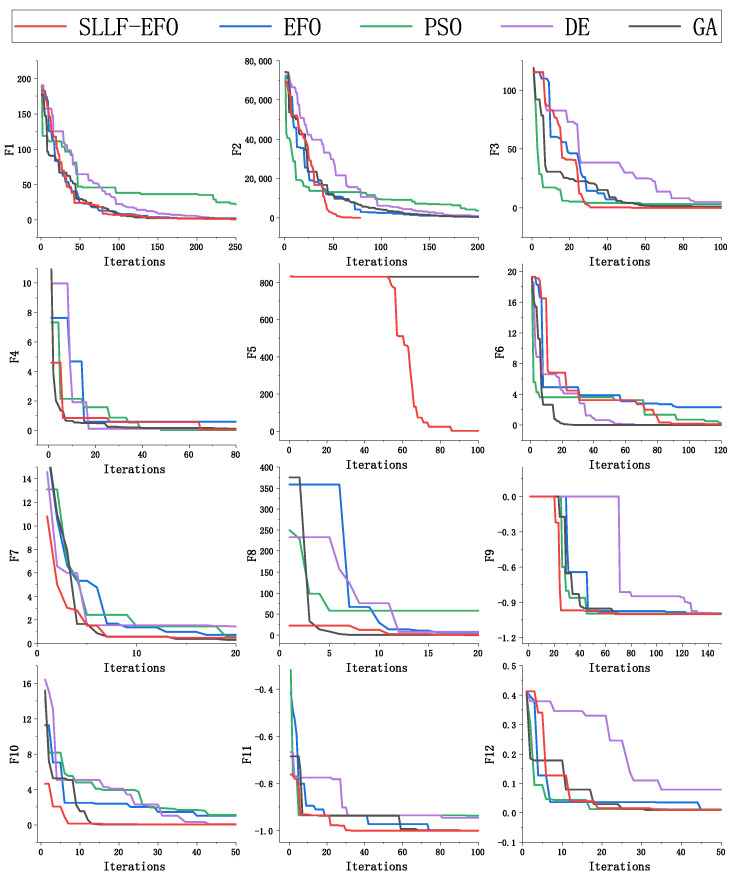
Convergence curves of 5 algorithms under 12 benchmark test functions.

The speed index results of the five algorithms conducting 100 experiments under different test functions are shown in Table 2. “Exceed” in this table indicates that the required number of iterations for the 100 experiments in this environment is greater than 2000. In addition to the three speed indicators, the last column in the table shows the ratio of the average iteration times of the EFO algorithm and the SLLF-EFO algorithm in all function environments that can successfully converge, which is the speed improvement factor (SIF) of the SLLF-EFO. And to visually demonstrate the convergence process of the algorithm, the parts of the iterative process with obvious convergence are shown and an image is drawn, as shown in Figure 5. These curves show the performance of the SLLF-EFO, EFO [9], PSO [43], DE [44], and GA [45] algorithms across 12 different benchmark functions. The X-axis represents the number of iterations, while the Y-axis represents the fitness value. The curves highlight the convergence speed of each algorithm, demonstrating the improved performance of the SLLF-EFO algorithm.

Based on Table 2 and Figure 5, the SLLF-EFO requires fewer iterations for optimization across 12 different test function environments compared to EFO. This suggests that the convergence speed of SLLF-EFO consistently outpaces that of EFO. Analysis of the GA data in the table reveals that the selected function tends to cause premature convergence and lead to the algorithm becoming trapped in a local optimum. In contrast, SLLF-EFO demonstrates the ability to achieve convergence within 2000 iterations across the majority of environments. This underscores the effectiveness of the improved algorithm in addressing the issue of stagnation encountered in the original algorithm.

Table 2 shows that the number of iterations of SLLF-EFO in the environment of 12 different test functions is less than that of EFO. This indicates that the convergence speed of SLLF-EFO is stable and faster than that of EFO. Observing the data of the GA algorithm in the table, it can be seen that the selected function easily leads to premature convergence and stagnation in local optimum of the algorithm. Observing the “Over-Num” metric, SLLF-EFO can converge within 2000 iterations in most environments, indicating that the improved algorithm effectively solves the problem of algorithm stagnation.

Observing the convergence curves of various algorithms in the early stages of iteration in Figure 5, it can be seen that under the F4, F7, F8, F10, and F11 functions, the initial fitness value of the SLLF-EFO is significantly better than the other four algorithms, which reduces the search difficulty of subsequent algorithms and fully verifies the optimization improvement of the good point set theory on population initialization.

In the F5 function environment depicted in Figure 5, all four algorithms, except for SLLF-EFO, initially converged to local optima. Consequently, they remained stuck in these local optimal solutions throughout the subsequent iterations, leading to premature convergence, as illustrated by a horizontal straight line in the figure. In contrast, the SLLF-EFO incorporates a standstill label strategy. This strategy involves monitoring the algorithm’s progress using a counter mechanism. Specifically, after detecting that it has reached a local optimum following 50 consecutive iterations, the algorithm activates a standstill label strategy. This strategy facilitates the escape from local optima, enabling individuals to explore the global search space. As evidenced by the convergence curve of F5, the SLLF-EFO algorithm exhibits a phase of horizontal convergence, followed by a resurgence in global search activity triggered by the standstill label strategy. This observation underscores the enhanced efficiency of the algorithm in seeking the global optimum through the implementation of the standstill label strategy.

Observing the optimization speed results of the SLLF-EFO and the other three meta-heuristic algorithms, it can be seen that the convergence speed of the SLLF-EFO is among the top performers in the five low-dimensional environments of F8-F12, and in these low-dimensional environments, the SLLF-EFO has the ability to find the global optimum. Except for the second place under the F9 function, all other environments only require the least number of iterations. In the high-dimensional complex environment of F1–F7, SLLF-EFO also maintains a high optimization efficiency, and the most obvious improvement is in the F2 function: in the F2 function environment, SLLF-EFO is ahead of the other four algorithms with a great advantage of finding the optimal value in an average of 112 iterations. In Figure 5, it can be seen that SLLF-EFO has the top convergence speed in most environments.

Under the conditions that SLLF-EFO can find the global optimum, observing the speed improvement multiple index, the algorithm in this paper requires fewer iterations than the EFO algorithm. This indicates that the improved SLLF-EFO algorithm has a high convergence speed in low-dimensional environments.

### 4.3. Accuracy Comparison with Other Algorithms

In addition to the study of algorithm search speed, the study of algorithm optimization quality is also very important. Under many functions or application conditions, meta-heuristic algorithms such as the EFO algorithm can only find approximate optimal values. Therefore, whether the algorithm can find an approximate value closer to the theoretical optimal value (i.e., accuracy) is particularly important. This article takes the best value (min), worst value (max), average (mean) and standard deviation (std) of 100 experimental test results as indicators to evaluate the search quality of the SLLF-EFO algorithm.

**Figure 6:**
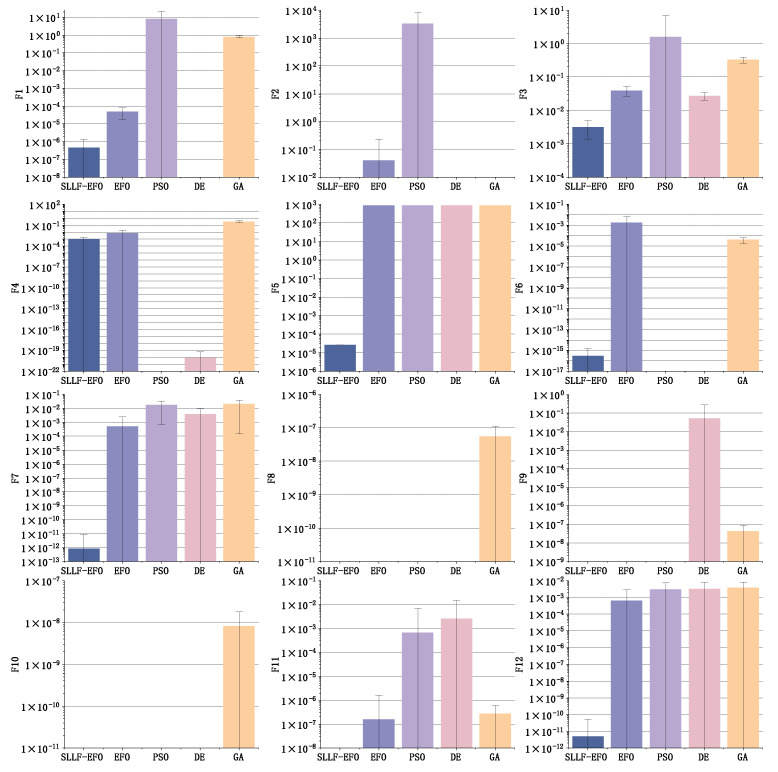
Error bands of search results for each algorithm under 12 standard test functions.

The error bands of 100 experimental results of the five algorithms under different test functions are shown in Figure 6. In Figure 6, to ensure consistency and facilitate comparison with other functions that have a theoretical optimum value of 0, we have made an adjustment to the presentation of the search results for functions F9 and F11, which theoretically have an optimum value of −1. Specifically, we have added 1 to the search results of these two functions, effectively shifting their optimum to 0. This uniform adjustment allows for a direct and apples-to-apples comparison across all benchmark functions depicted in the figure, without loss of generality or accuracy in the representation of the algorithms’ performance.

The bands in Figure 6 illustrate the stability and robustness of each algorithm, with narrower bands indicating more consistent performance. A comparison of search results between the SLLF-EFO algorithm and the other algorithms under each function is shown in Table 3. The last column in the table calculates the ratio of various indicators of the EFO algorithm and SLLF-EFO algorithm in all function environments where the optimal solution cannot be successfully found, which is conducive to demonstrating the optimization ability and stability improvement of the SLLF-EFO algorithm compared to the EFO algorithm.

In Figure 6, we establish a convention: if an algorithm can consistently find the optimal value on a specific test function, i.e., the “Mean” metric in Table 3 is 0, then it does not display a bar height in Figure 6, which uses a logarithmic coordinate system, while all other non-zero data are displayed. This approach not only fully reflects the advantage of algorithms that can stably converge to the global optimum but also more clearly highlights the differences among algorithms that cannot consistently find the optimal value.

**Figure 7:**
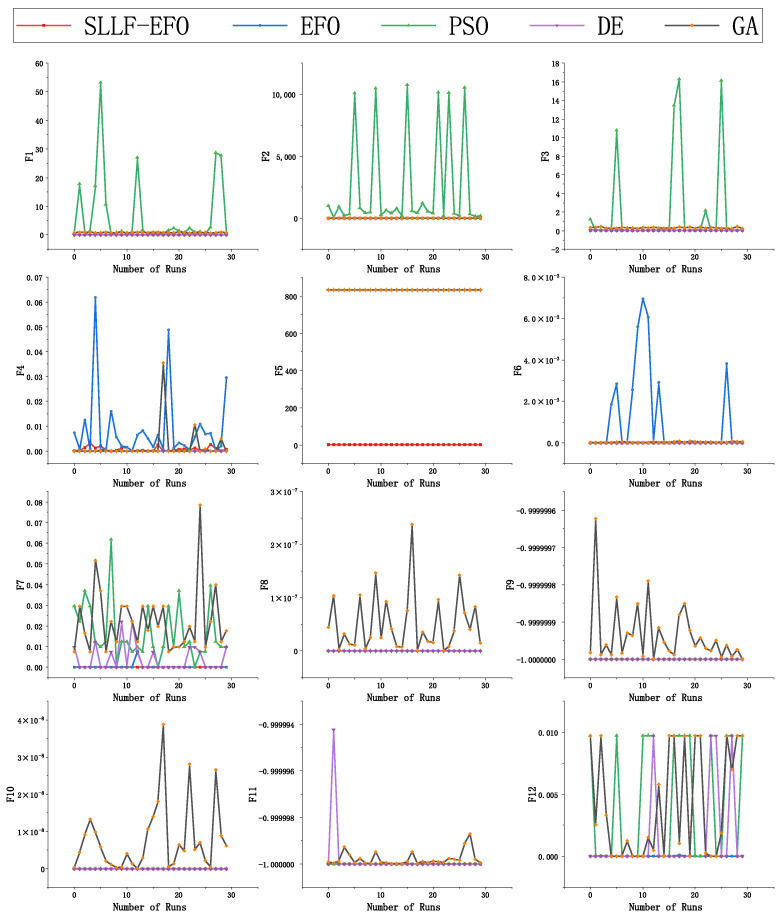
Thirty repeated experimental test results.

Moreover, in order to more intuitively demonstrate the search stability of the five algorithms, 30 randomly selected test results from 100 experiments were plotted as a line graph, as shown in Figure 7. The X-axis represents the number of runs, and the Y-axis represents the fitness values. The plot highlights the variability in performance and the consistency of the SLLF-EFO algorithm compared to the others.

According to Figure 6 and Table 3, it can be seen that under 12 benchmark function environments, the optimization results of the SLLF-EFO algorithm are also significantly better than those of the EFO algorithm, and the theoretical optimal values can be stably searched in the F2, F8, F9, F10, and F11 environments. In the low-dimensional environments of F8–F12, the optimization results of the SLLF-EFO algorithm are stable and superior to the other three algorithms, which fully demonstrates the optimization adaptability of the SLLF-EFO algorithm in low-dimensional environments. In the high-dimensional environments of F1–F7, except for a few cases (such as F4), the SLLF-EFO algorithm can also achieve high accuracy.

The results of the F5 function in Table 3 show that the convergence results of the other four algorithms are the same, with a standard deviation of 0, indicating that in 100 repeated experiments, the F5 function caused all algorithms to quickly fall into local optima in the early iterations. However, the SLLF-EFO algorithm relied on the standstill label strategy to jump out of the initial local optimum and ultimately obtained quite good search results. The other four algorithms, due to the lack of solutions against falling into local optimum, always stagnated at the initial local optimum and did not move, resulting in unsatisfactory search results. This further demonstrates the advantages of the standstill label strategy in this paper.

By comparing the ratio data of the “Mean” metric between the SLLF-EFO algorithm and the original EFO algorithm, the experimental results of the SLLF-EFO algorithm show a stable improvement compared to the EFO algorithm, with a minimum ratio of 8.62 (F4). In the F6 and F7 environments, the average fitness value of the SLLF-EFO algorithm increases by 10 and 8 orders of magnitude, respectively, compared to the EFO algorithm.

Observing the two indexes of “Min” and “Max” in Table 3, in all 12 function environments, the SLLF-EFO algorithm’s two indicators are not lower than those of the EFO algorithm. The minimum accuracy obtained by the SLLF-EFO algorithm in 100 experiments is at least 2.62 times (F4) that of the EFO algorithm, while the maximum accuracy is at least 9.03 times (F3). This indicates that the SLLF-EFO algorithm has lower errors than the EFO algorithm, further demonstrating its higher stability and ability to find better quality positions than the EFO algorithm. This once again proves the advantages of the improved scheme proposed in this article.

From Figure 6, it is evident that both the optimization results and the standard deviation of the SLLF-EFO algorithm are at a relatively low level. Observing Figure 7, among the 30 randomly selected test results, the PSO algorithm and GA algorithm exhibit strong instability in most cases, while the EFO algorithm and DE algorithm also exhibit instability in some functions. The fluctuation line of the SLLF-EFO algorithm is always smooth and has strong robustness, which also confirms the error band distribution in Figure 6 and is reflected in the std ratio indicator in Table 3. Looking only at the function environment that has not reached the global optimum, the standard deviation ratio between the EFO algorithm and SLLF-EFO algorithm is still the lowest at 6.98 (F3), and it exhibits extremely high stability compared to EFO in the F6, F7, and F12 environments. This fully indicates that the improved SLLF-EFO algorithm greatly improves the global optimization effect and stability of the EFO algorithm.

This paper selected the average “Mean” of the optimization results of all five algorithms under 12 test functions for ranking, and took the average of the rankings for data with the same ranking, as shown in Table 4. These “Mean” values were used as a hypothesis test to implement the Friedman test [48].

According to Table 4, we can see that the SLLF-EFO algorithm can achieve good rankings in various function environments, and the ranking obtained in all 12 functions is not lower than that of the original EFO algorithm. We believe that the null hypothesis $H_{0}$ means that there is no difference in the performance of each algorithm. As an alternative hypothesis, we believe that $H_{1}$ represents the performance differences among various algorithms. The significance level in this test hypothesis is α=0.05. When *p*-value < α, we reject $H_{0}$ and calculate the Friedman value $\tau_{f}$ to be 15.58209 and the *p*-value to be 3.3634 $\times \;10^{-3}$. Therefore, we reject $H_{0}$, which proves that the SLLF-EFO algorithm is significantly superior to other algorithms.

To provide further evidence of the enhanced performance of the SLLF-EFO algorithm, an additional Wilcoxon signed-rank test was conducted on the “mean” values obtained from the SLLF-EFO and the original EFO algorithms. This non-parametric statistical test is appropriate for assessing the difference in central tendencies between two related samples [49], in this instance, the mean performance values of the two algorithms. The Wilcoxon signed-rank test yielded a *p*-value of 7.6858 $\times \;10^{-3}$, which falls below the traditional significance threshold of 0.05. This statistically significant result indicates a notable difference between the mean performances of the SLLF-EFO and EFO algorithms, suggesting that the SLLF-EFO algorithm demonstrates superior solution quality. The low *p*-value implies that the observed improvement in the mean performance of the SLLF-EFO algorithm is highly unlikely to be a result of random variation, thereby reinforcing the conclusion that the refinements incorporated into the SLLF-EFO algorithm have led to a marked enhancement in optimization efficacy.

## 5. Conclusions

As a new type of meta-heuristic algorithm, the optimization exploration of the EFO algorithm holds significant research value and reference importance. This article analyzes and tests the EFO algorithm, identifying issues such as premature convergence in complex environments, and proposes innovative solutions to these problems. The proposed SLLF-EFO algorithm integrates optimization techniques from multiple EFO algorithms, such as the theory of good point sets, a variable-step-size Levy flight strategy, and a stagnation marking strategy, to systematically enhance the optimization performance of the EFO algorithm.

The analysis of simulation test results based on standard test functions reveals that the SLLF-EFO algorithm has a substantially improved convergence speed, accuracy, and stability compared to the EFO algorithm. The enhanced convergence speed of the SLLF-EFO can be attributed to its use of a good point set for population initialization, ensuring an even distribution of the initial population across the entire search space. Additionally, the algorithm employs an adaptive global range strategy that rapidly reduces the search area, facilitating the identification of the approximate direction of the global optimal solution during the global search phase.

Regarding solution accuracy and stability, the SLLF-EFO incorporates an optimized individual selection strategy for active electric field localization, enhancing the uniformity of population distribution throughout the iteration process. In the passive electric field localization phase, the algorithm integrates the Levy flight search strategy with a variable step size, increasing the algorithm’s stochasticity and jumping ability, addressing problem 3 as outlined in Section 2.2. Furthermore, the introduction of the golden sine strategy enhances population diversity, while the use of the standstill label strategy reduces the number of iterations and facilitates escaping local optima, effectively resolving problems 1 and 2 highlighted in Section 2.2.

Consequently, the SLLF-EFO demonstrates robust global search capability and adaptability to various environments, outperforming the EFO in terms of solution accuracy and stability, showing its superiority over other meta-heuristic algorithms in diverse settings.

This article addresses the optimization of the EFO algorithm with the objective of resolving the issue of passive electric field localization stagnation. It also enhances the optimization capability of the EFO algorithm, enabling it to effectively navigate diverse and complex environmental conditions. Potential future research directions include exploring the integration of other meta-heuristic algorithms to further enhance the performance of the EFO algorithm or applying it to complex optimization challenges in various engineering projects. In conclusion, the optimization of the EFO algorithm discussed in this article has substantial implications for guiding future research endeavors involving the EFO algorithm.

## Figures and Tables

**Table 1:** Benchmark test functions.

Serial Number	Function	Search Scope	Dimension	Optimum Value
F1	Sphere	[−5.12, 5.12]	30	0
F2	Step	[−100, 100]	30	0
F3	Quartic	[−1.28, 1.28]	30	0
F4	Rosenbrock	[−5, 10]	30	0
F5	Schwefel	[−500, 500]	30	0
F6	Ackley	[−32, 32]	30	0
F7	Griewank	[−600, 600]	30	0
F8	Bohachevsky	[−100, 100]	2	0
F9	Easom	[−100, 100]	2	−1
F10	Rastrigin	[−5.12, 5.12]	2	0
F11	Drop-Wave	[−5.12, 5.12]	2	−1
F12	Schaffer N6	[−10, 10]	2	0

**Table 2:** Comparison of speeds of various algorithms under 12 functions.

Function	Index	SLLF-EFO	EFO	PSO	DE	GA	SIF
F1	—	Exceed	Exceed	Exceed	Exceed	Exceed	—
F2	Min-Iter	62	1005	2000	481	361	13.64
	Max-Iter	441	2000	2000	572	827	
	Mean-Iter	112.17	1530.15	2000	540.34	502.61	
	Over-Num	0	4	100	0	0	
F3	—	Exceed	Exceed	Exceed	Exceed	Exceed	—
F4	Min-Iter	2000	2000	831	2000	2000	—
	Max-Iter	2000	2000	1291	2000	2000	
	Mean-Iter	2000	2000	1009.67	2000	2000	
	Over-Num	100	100	0	100	100	
F5	—	Exceed	Exceed	Exceed	Exceed	Exceed	—
F6	Min-Iter	730	938	797	379	2000	1.57
	Max-Iter	2000	2000	1158	464	2000	
	Mean-Iter	994.52	1563.65	1005.98	422.67	2000	
	Over-Num	6	31	0	0	100	
F7	Min-Iter	327	781	804	410	2000	1.32
	Max-Iter	2000	2000	2000	2000	2000	
	Mean-Iter	1350.48	1789.35	1875.16	1093.83	2000	
	Over-Num	13	68	88	40	100	
F8	Min-Iter	109	157	514	207	2000	1.90
	Max-Iter	139	801	1074	283	2000	
	Mean-Iter	122.51	223.23	798.59	250.04	2000	
	Over-Num	0	0	0	0	100	
F9	Min-Iter	375	803	529	259	2000	2.33
	Max-Iter	1483	1970	1007	2000	2000	
	Mean-Iter	596.18	1388.08	773.71	431.93	2000	
	Over-Num	0	0	0	5	100	
F10	Min-Iter	105	187	517	181	2000	2.00
	Max-Iter	229	756	1054	260	2000	
	Mean-Iter	140.89	281.13	761.67	226.42	2000	
	Over-Num	0	0	0	0	100	
F11	Min-Iter	162	403	506	371	2000	3.30
	Max-Iter	582	2000	2000	2000	2000	
	Mean-Iter	263.02	868.33	771.86	550.53	2000	
	Over-Num	0	10	1	4	100	
F12	Min-Iter	302	931	585	510	2000	2.38
	Max-Iter	2000	2000	2000	2000	2000	
	Mean-Iter	796.27	1894.89	1218.83	1263.89	2000	
	Over-Num	4	72	31	33	100	

**Table 3:** Search results for 12 types of functions.

Function	Index	SLLF-EFO	EFO	PSO	DE	GA	Ratio
F1	Min	1.86356 $\times \;10^{-11}$	6.05359 $\times \;10^{-6}$	7.46691$\times \;10^{-3}$	5.14144 $\times \;10^{-17}$	4.29041 $\times \;10^{-1}$	3.25 $\times \;10^{5}$
	Max	6.17920 $\times \;10^{-6}$	1.34577 $\times \;10^{-4}$	5.734946 $\times \;10^{1}$	1.07362 $\times \;10^{-15}$	1.022556 $\times \;10^{1}$	21.78
	Mean	4.60575 $\times \;10^{-7}$	4.85313 $\times \;10^{-5}$	8.078069	2.45612 $\times \;10^{-16}$	8.01901 $\times \;10^{-1}$	105.37
	Std	9.74995 $\times \;10^{-7}$	3.05088 $\times \;10^{-5}$	1.408101 $\times \;10^{1}$	1.89315 $\times \;10^{-16}$	1.16547 $\times \;10^{-1}$	31.29
F2	Min	0	0	31	0	0	—
	Max	0	1	2.0269 $\times \;10^{4}$	0	0	INF
	Mean	0	0.04	3.306320 $\times \;10^{3}$	0	0	INF
	Std	0	1.96946 $\times \;10^{-1}$	5.036425 $\times \;10^{3}$	0	0	INF
F3	Min	4.70747 $\times \;10^{-5}$	1.13327 $\times \;10^{-2}$	3.69332 $\times \;10^{-4}$	1.36835 $\times \;10^{-2}$	1.27985 $\times \;10^{-1}$	240.74
	Max	9.13077 $\times \;10^{-3}$	8.24585 $\times \;10^{-2}$	3.76160 $\times \;10^{1}$	4.20763 $\times \;10^{-2}$	4.87395 $\times \;10^{-1}$	9.03
	Mean	3.14774 $\times \;10^{-3}$	3.89538 $\times \;10^{-2}$	1.58296	2.69964 $\times \;10^{-2}$	3.26350 $\times \;10^{-1}$	12.38
	Std	1.78131 $\times \;10^{-3}$	1.24362 $\times \;10^{-2}$	5.36132	6.63866 $\times \;10^{-3}$	6.72907 $\times \;10^{-2}$	6.98
F4	Min	1.95206 $\times \;10^{-5}$	5.10639 $\times \;10^{-5}$	0	6.79406 $\times \;10^{-27}$	1.32225 $\times \;10^{-1}$	2.62
	Max	4.26540 $\times \;10^{-3}$	6.63270 $\times \;10^{-2}$	0	5.45878 $\times \;10^{-19}$	5.02718 $\times \;10^{-1}$	15.55
	Mean	8.54922 $\times \;10^{-4}$	7.36785 $\times \;10^{-3}$	0	7.45493 $\times \;10^{-21}$	3.19381 $\times \;10^{-1}$	8.62
	Std	8.84378 $\times \;10^{-4}$	1.29638 $\times \;10^{-2}$	0	5.48703 $\times \;10^{-20}$	7.32088 $\times \;10^{-2}$	14.66
F5	Min	2.54551 $\times \;10^{-5}$	8.318099 $\times \;10^{2}$	8.318099 $\times \;10^{2}$	8.318099 $\times \;10^{2}$	8.318099 $\times \;10^{2}$	3.27 $\times \;10^{7}$
	Max	2.54551 $\times \;10^{-5}$	8.318099 $\times \;10^{2}$	8.318099 $\times \;10^{2}$	8.318099 $\times \;10^{2}$	8.318099 $\times \;10^{2}$	3.27 $\times \;10^{7}$
	Mean	2.54551 $\times \;10^{-5}$	8.318099 $\times \;10^{2}$	8.318099 $\times \;10^{2}$	8.318099 $\times \;10^{2}$	8.318099 $\times \;10^{2}$	3.27 $\times \;10^{7}$
	Std	2.26102 $\times \;10^{-14}$	0	0	0	0	—
F6	Min	0	0	0	0	3.58210 $\times \;10^{-6}$	—
	Max	7.10543 $\times \;10^{-15}$	3.59945 $\times \;10^{-2}$	0	0	9.50181 $\times \;10^{-5}$	5.07 $\times \;10^{12}$
	Mean	2.84217 $\times \;10^{-16}$	1.76731 $\times \;10^{-3}$	0	0	3.99185 $\times \;10^{-5}$	6.22 $\times \;10^{10}$
	Std	1.20346 $\times \;10^{-15}$	5.21965 $\times \;10^{-3}$	0	0	2.27945 $\times \;10^{-5}$	4.33 $\times \;10^{12}$
F7	Min	0	0	0	0	2.62129 $\times \;10^{-7}$	—
	Max	8.35549 $\times \;10^{-11}$	9.85816 $\times \;10^{-3}$	7.63995 $\times \;10^{-2}$	2.95866 $\times \;10^{-2}$	1.36342 $\times \;10^{-1}$	1.18 $\times \;10^{8}$
	Mean	8.43037 $\times \;10^{-13}$	5.04985 $\times \;10^{-4}$	1.74564 $\times \;10^{-2}$	3.69777 $\times \;10^{-3}$	1.99633 $\times \;10^{-2}$	5.99 $\times \;10^{8}$
	Std	8.35483 $\times \;10^{-12}$	1.88671 $\times \;10^{-3}$	1.68058 $\times \;10^{-2}$	5.27329 $\times \;10^{-3}$	1.98104 $\times \;10^{-2}$	2.26 $\times \;10^{8}$
F8	Min	0	0	0	0	2.25695 $\times \;10^{-11}$	—
	Max	0	0	0	0	2.93853 $\times \;10^{-7}$	—
	Mean	0	0	0	0	5.41664 $\times \;10^{-8}$	—
	Std	0	0	0	0	5.76108 $\times \;10^{-8}$	—
F9	Min	−1	−1	−1	−1	−0.999999999	—
	Max	−1	−1	−1	0	−0.999999798	—
	Mean	−1	−1	−1	−0.95	−0.999999957	—
	Std	0	0	0	2.19043 $\times \;10^{-1}$	4.36475 $\times \;10^{-8}$	—
F10	Min	0	0	0	0	2.25695 $\times \;10^{-11}$	—
	Max	0	0	0	0	5.49201 $\times \;10^{-8}$	—
	Mean	0	0	0	0	8.15796 $\times \;10^{-9}$	—
	Std	0	0	0	0	9.64681 $\times \;10^{-9}$	—
F11	Min	−1	−1	−1	−1	−0.999999999	—
	Max	−1	−0.999985339	−0.936245328	−0.936245328	−0.999997414	INF
	Mean	−1	−0.999999843	−0.999362453	−0.997449813	−0.999999729	INF
	Std	0	1.46723 $\times \;10^{-6}$	6.37547 $\times \;10^{-3}$	1.25563 $\times \;10^{-2}$	3.38904 $\times \;10^{-7}$	INF
F12	Min	0	0	0	0	3.15666 $\times \;10^{-9}$	—
	Max	4.70372 $\times \;10^{-10}$	9.71591 $\times \;10^{-3}$	9.71591 $\times \;10^{-3}$	9.71591 $\times \;10^{-3}$	9.71591 $\times \;10^{-3}$	2.07 $\times \;10^{7}$
	Mean	4.71959 $\times \;10^{-12}$	6.14620 $\times \;10^{-4}$	3.01193 $\times \;10^{-3}$	3.20625 $\times \;10^{-3}$	3.81969 $\times \;10^{-3}$	1.30 $\times \;10^{8}$
	Std	4.70359 $\times \;10^{-11}$	2.19889 $\times \;10^{-3}$	4.51618 $\times \;10^{-3}$	4.59156 $\times \;10^{-3}$	4.32392 $\times \;10^{-3}$	4.67 $\times \;10^{7}$

**Table 4:** Ranking table of 5 algorithms.

Serial Number	SLLF-EFO	EFO	PSO	DE	GA
F1	2	3	5	1	4
F2	2	5	4	2	2
F3	1	3	5	2	4
F4	3	4	1	2	5
F5	1	3.5	3.5	3.5	3.5
F6	3	5	1.5	1.5	4
F7	1	2	4	3	5
F8	2.5	2.5	2.5	2.5	5
F9	2	2	2	4	5
F10	2.5	2.5	2.5	2.5	5
F11	1	2	4	5	3
F12	1	2	3	4	5

## Data Availability

Data are contained within the article.
